# Exploration of Automated Measurement for Ossicular Chains Based on 3-Dimensional Geometric Information

**DOI:** 10.34133/cbsystems.0305

**Published:** 2025-07-02

**Authors:** Mengshi Zhang, Yufan Zhang, Sihui Guo, Xiaoguang Li, Li Zhuo, Yuxue Ren, Wei Chen, Yili Feng, Ruowei Tang, Han Lv, Pengfei Zhao, Zhenchang Wang, Hongxia Yin

**Affiliations:** ^1^ Department of Radiology, Beijing Friendship Hospital, Capital Medical University, Beijing 100050, China.; ^2^ Beijing Key Laboratory of Computational Intelligence and Intelligent System, Beijing University of Technology, Beijing 100124, China.; ^3^ Academy for Multidisciplinary Studies, Capital Normal University, Beijing 100048, China.; ^4^ Department of Medical Engineering, Beijing Friendship Hospital, Capital Medical University, Beijing 100050, China.; ^5^ Department of Automation, Tsinghua University, Beijing 100084, China.

## Abstract

Abnormalities in the ossicular chain, a key middle-ear component that is crucial for sound transmission, can lead to conductive hearing loss; reconstruction offers an effective treatment. Accurate preoperative ossicular-chain measurements are essential for creating prostheses; however, current methods rely on cadaver studies or manual measurements from 2-dimensional images, which are time-intensive and laborious and depend heavily on radiologist expertise. To improve efficiency, we aimed to develop a systematic approach for automated ossicular-chain segmentation and measurement using ultra-high-resolution computed tomography (U-HRCT). One hundred forty patients (226 ears) with normal ear anatomy underwent U-HRCT. Twelve parameters were defined to measure ossicular-chain components. Automated measurements based on automated segmentation of 226 ear images were verified through manual measurements. We analyzed variations by ear side, sex, and age group. Stapes analysis was limited by segmentation accuracy. Complete segmentation of the malleus, incus, and stapes was achieved in 47 ears. Automated measurements of 8 parameters showed no significant differences compared to manual measurements in 47 cases. Significant sex-based differences emerged in all parameters except stapes footplate length, incudostapedial joint angle, and stapes volume (*P* = 0.205, *P* = 0.560, and *P* = 0.170, respectively). Notable side-specific differences were observed in female incus height and male malleus volume (*P* = 0.017 and *P* = 0.037, respectively). No statistically significant differences were found in other parameters across different age groups, except for malleus and incus volumes (*P* = 0.015 and *P* = 0.031). The proposed algorithm effectively automated ossicular-chain segmentation and measurement, establishing a normative range for ossicular parameters and providing a valuable reference for detecting abnormalities.

## Introduction

The ossicular chain, consisting of the malleus, incus, and stapes, transmits acoustic vibrations from the outer to the inner ear, enabling sound perception. Abnormalities in any part of the ossicular chain can disrupt sound conduction and may result in hearing loss [1,2]. Middle ear conditions such as otitis media [3], cholesteatoma [4,5], and trauma [6] can impair the ossicular chain’s integrity and continuity, often leading to conductive hearing loss. Ossicular-chain reconstruction surgery is an effective intervention [7–9] that can restore sound transmission in the middle ear and improve hearing. Accurate preoperative measurements of the ossicular chain are essential for preparing personalized ossicular-chain prostheses for patients undergoing this procedure.

Computed tomography (CT) is widely used for diagnosing middle ear lesions. However, conventional high-resolution CT often fails to fully visualize the stapes owing to its limited resolution [10]. The introduction of ultra-high-resolution computed tomography (U-HRCT) has significantly enhanced the imaging of temporal bones, enabling clearer visualization of delicate structures and hidden lesions [11]. The U-HRCT scanner developed by our research team has extremely high spatial resolution, which can reach up to 0.05 mm. It is capable of clearly showing the fine structures of the ossicular chain, and the visualization rate of anatomical structures such as the stapes footplate can reach 100% [12]. This advancement provides a high-quality data foundation for precise segmentation and is becoming a valuable tool in ossicular-chain imaging and reconstructive surgery. Although U-HRCT provides radiologists with high-quality data, the measurement of the ossicular chain still relies on manual measurement. This method not only is time-consuming and labor-intensive but also makes it impractical to measure a large volume of data. Consequently, further research is necessary to investigate how to leverage high-quality data for a quantitative analysis of fully automated measurements of the ossicular chain.

Most ossicular-chain measurements in current research rely on tools like calipers or optical microscopes on cadaveric specimens or during surgery. For instance, Javia and Saravanan [13] examined 60 dry adult mallei, comparing lengths in male and female cadavers. Singh et al. [14] measured ossicular integrity by conducting cortical mastoidectomy on 47 cadaveric temporal bones, using microscopy and Digimizer software to capture images for anatomical measurement. These methods, although detailed, are unsuitable for in vivo assessment, limiting their application in preoperative planning. Some studies have used software for manual 2-dimensional (2D) CT measurements. Akazawa et al. [11] semiautomatically measured stapes footplate thickness, whereas Rousset et al. [15] manually measured key stapes dimensions on 2D CT images. However, manual measurement is time-consuming and laborious and often requires multiple measurements to ensure reliability. The accuracy of these measurements depends heavily on radiologist expertise. Due to the varying diagnostic experiences of physicians in clinical practice, the absence of standardized training and unified measurement criteria can lead to inconsistent measurement results.

Automated ossicular-chain measurements could overcome these limitations by improving accuracy, repeatability, and efficiency, thus reducing the workload of radiologists. Despite these potential benefits, automated ossicular-chain measurement remains underexplored. Ding et al. [16] used template-based segmentation on 52 CBCT (cone beam computed tomography) images of temporal bones, automatically measuring malleus, incus, and facial nerve lengths and angles. This method [17] relied on a pre-annotated temporal bone atlas, with image registration techniques aligning the atlas to each target image to map annotations. Although effective, this approach depends on an average temporal bone template and requires a substantial number of CT images to generate an accurate template. Its limitations may lead to inaccuracies in segmenting and measuring atypical or anatomically varied images. Additionally, this method did not address measurements of the stapes, the smallest bone in the body, and a critical component of sound transmission, connecting the oval window between the incus bone and the inner ear. Accurate measurement of stapes parameters is essential for effective preoperative planning, but detailed anatomical data on ossicular chains in living humans remain scarce. The tiny and delicate structure of the ossicular chain makes the accurate detection of hidden lesions challenging.

In this study, we propose an automated measurement method that integrates a surrounding box algorithm with 3D geometric data from the ossicular chain, building on previous work with deep learning algorithms. This novel method enables direct automated measurement of the 3D ossicular chain and successfully achieves the automatic measurement of 12 parameters of the ossicular chain. When applied clinically, it can provide a rapid and effective tool for the automated analysis of large amounts of data, addressing the issue of inconsistent measurement results that arise from the absence of standardized training in manual measurement. In the surface reconstruction stage, this study employs the existing Ricci curvature level set method to refine the segmentation results at a subvoxel level and utilizes the Marching Cubes algorithm to generate a triangular mesh model. Building on this, a novel approach is introduced by incorporating a windowed sinc function interpolation kernel for low-pass filtering and surface smoothing, effectively suppressing high-frequency noise while preserving the geometric details of key anatomical features to the greatest extent. In the geometric measurement stage, this study innovatively combines curvature analysis with the minimum bounding box to achieve accurate detection of target feature points. Furthermore, to address the complex morphology of the stapes footplate, a major axis estimation method is designed to optimize feature point localization and improve positioning accuracy. This study successfully achieved the automatic measurement of the 3D structure of the ossicular chain and has been applied with preliminary exploration in a relatively large dataset of 226 normal ears. It established normal value ranges for 12 measurement parameters, providing valuable data references for diagnosing ossicular-chain abnormalities. Furthermore, the study analyzed differences in measurement results across various genders, sides, and age groups, offering important insights into the biological variations of the ossicular chain among different populations.

## Materials and Methods

### Subjects and CT scans

This study adheres to the Declaration of Helsinki (revised in 2013) and received approval from the Ethics Committee of Beijing Friendship Hospital, Capital Medical University (IRB: 2024-P2-209), with the requirement for informed consent waived due to the retrospective nature of the study. We included patients who underwent U-HRCT between July 2021 and September 2023. Inclusion criteria were as follows: (a) U-HRCT showed no notable abnormalities in the malleus, incus, stapes, or their spatial relationships; and (b) patients were 18 years or older. Exclusion criteria included (a) ear deformity, trauma, tumors, or inflammation; (b) history of ontological surgery; and (c) significant artifacts compromising image quality. Since all the included subjects were patients examined in the hospital, it is possible that only one ear of each patient was normal. Therefore, a total of 226 normal ears from 140 patients were finally included.

Each patient underwent unilateral U-HRCT using a U-HRCT scanner (LargeV Instrument Corp., Beijing, China) with settings of 100- to 110-kVp voltage, 120- to 180-mAs current, and a 65-mm field of view. Moreover, section thickness and interval were set to 0.1 mm. Isotropic axial images were acquired 20 s after scanning.

### Process of manual annotation

First, an annotation team composed of medical imaging professionals was established, with all members possessing expertise in medical imaging. Subsequently, experts with rich experience in ossicular-chain annotation conducted training sessions. After the training, the annotators performed single-blind annotations without access to the patients’ clinical information. They independently utilized Mimics software (Materialise, Belgium) to manually annotate the malleus, incus, and stapes in the U-HRCT images. Finally, experienced annotators conducted a single-blind review of the annotation results.

### Automated segmentation of the ossicular chains using TransUnet neural network

Accurate segmentation is essential for precise measurements. Our team has developed a deep learning algorithm utilizing multi-perspective fusion and active contour constraints for automated ossicular-chain segmentation in U-HRCT [18].

This method employs the TransUnet [19] architecture as its backbone, which is a hybrid encoder–decoder network that combines a transformer and convolutional neural network (CNN). The architecture of TransUnet employs a CNN-Transformer hybrid model where CNN is first used as a feature extractor to generate a feature map for the input. The source is provided in Ref. [19].

To maximize complementary information across views, a multi-perspective fusion algorithm was implemented to segment and combine ossicular chains from coronal, sagittal, and transverse sections, enhancing segmentation accuracy. To address challenges in segmenting the stapes, we used an active contour loss method based on the Euler curve energy, which constrains segmentation by average curvature and contour length, significantly improving stapes segmentation accuracy and integrity. Specifically, the totle loss of the segmentation model is shown in Eq. (1).

loss=0.5×Diceloss+0.5×CEloss+0.00001×ACEloss (1)

where the 3 terms are dice loss, cross entropy loss, and the active contour loss. *ACEloss* is shown in Eqs. (2) and (3).

$$\text{ACEloss}=\int \left(\alpha +\beta \cdot {\text{curvature}}^{2}\right)\text{length}$$ (2)

$$\text{curvature}=\frac{\left(1+u_{x}^{2}\right)u_{yy}+\left(1+u_{y}^{2}\right)u_{xx}-2u_{x}u_{y}u_{xy}}{2{\left(1+u_{x}^{2}+u_{y}^{2}\right)}^{\frac{3}{2}}}$$ (3)

where $u_{x}$, $u_{\mathrm{xx}}$, $u_{y}$, $u_{\mathrm{xy}}$, and $u_{\mathrm{yy}}$ are the discrete form of the central finite difference, and α=0.1, β=0.1 are the penalty coefficients of the contour’s length and curvature. The core idea is to constrain the contour with average curvature and length, thereby making the segmented target’s contour smoother.

Segmentation training was optimized with a combination of Dice loss and cross-entropy loss. Note that we have not included the training process for segmentation network. These problems have been addressed in Ref. [18]. For training details, we suggest to inquire the corresponding author in Ref. [18].

These advancements resolved challenges associated with small ossicular targets and data availability for network training. In the field of medical image segmentation, the dice similarity coefficient (DSC) is a critical metric for evaluating segmentation quality. It accurately quantifies the degree of overlap between automatically generated segmentation results and manual annotations. A higher DSC value signifies a greater level of agreement between the automatic segmentation output and the manual annotations. In other words, a higher DSC indicates a more precise and superior segmentation result. The segmentation accuracy for the malleus and incus was high, with average DSC of 94.2% and 94.6%, respectively, whereas stapes segmentation accuracy averaged a DSC of 76.4% [18].

TransUnet [19] is a popular architecture for medical image segmentation, which is an improved version of Unet. It introduces multiple Transformer blocks to enhance the ability of modeling long-range information. It verified that TransUnet outperforms the standard Unet on the Synapse multi-organ CT dataset. The average DSC is increased by about 3%. DeepLab models [20–22] are designed for segmenting natural images. They are not as popular as Unet in medical image segmentation tasks. We compared our method with 3D-DSD [23] and SwinUnet [24] in Ref. [18]. The results show that TransUnet outperforms the comparison methods.

No additional sequence preprocessing steps are required. In Ref. [18], preprocessing steps are included. Specifically, in order to improve the speed of ossicular-chain segmentation, ROI (region of interest) region positioning and cropping are used to crop the spatial resolution of U-HRCT images of 650 × 650 to 224 × 224 voxels.

Although we have introduced the active contour loss based on the Euler curve energy to improve stapes segmentation accuracy and integrity, the stapes is not as good at segmentation as other structures. Topology prior may provide additional supervised information for segmentation model. We will release the updated segmentation methods for completed stapes in the near future.

This laid the groundwork for further quantitative analysis. Following automated segmentation, results were manually verified by a radiologist who compared them with 2D CT images and 3D reconstructions, ultimately selecting images with complete and accurate ossicular-chain segmentation.

### Automated measurement of the ossicular chains using 3D geometric information

**Fig. 1.**
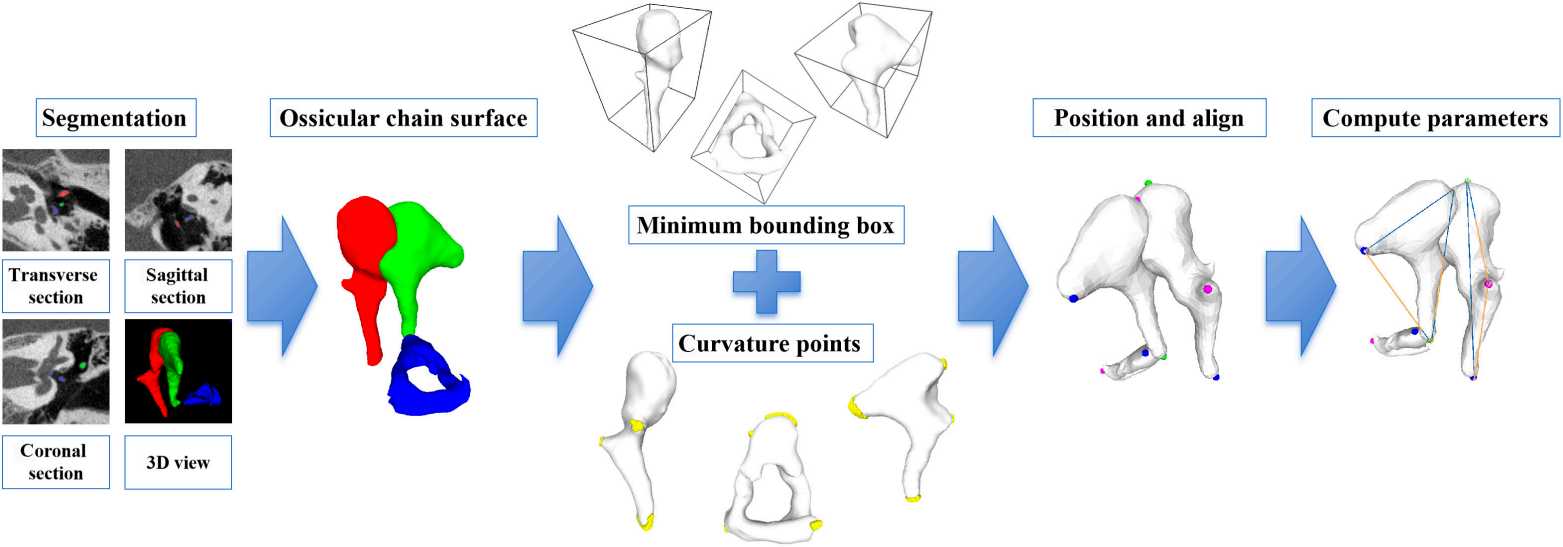
Automated measurement process of ossicular chain. The segmented red, green, and blue areas are the malleus, incus, and stapes, respectively.

This study proposes an automated algorithm for ossicular-chain measurement based on the 3D reconstruction of the 3 auditory ossicles. The algorithm identifies key feature points on the reconstructed grid, enabling the measurement of parameters based on these points. The process flowchart is depicted in Fig. 1.

**Fig. 2.**
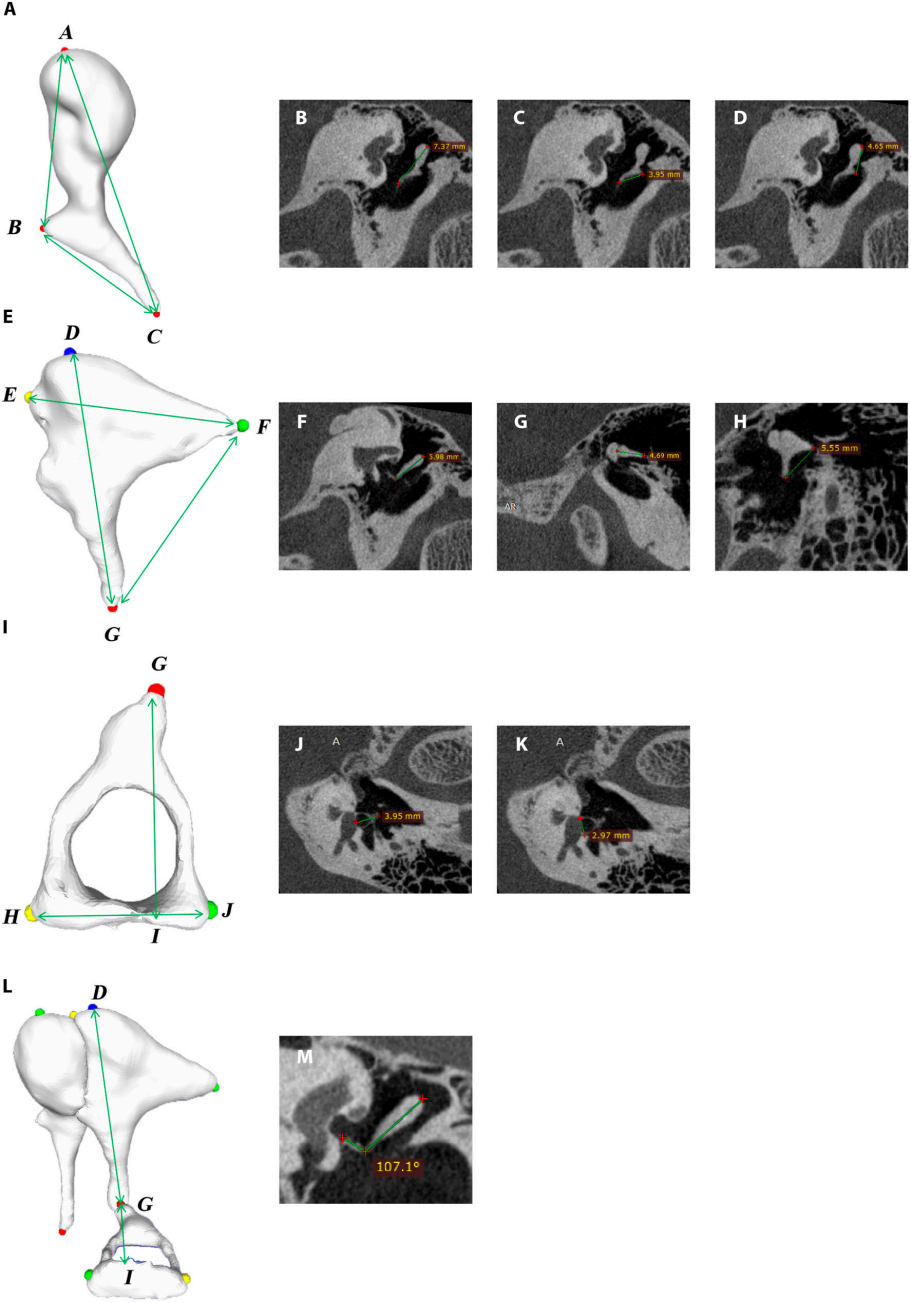
Automated measurement parameters of the ossicles chain. (A) *AC* is the total height of the malleus, *BC* is the length of the manubrium of the malleus, and *AB* is the length of the head and neck. (B to D) Manual measurement of the total height of the malleus, the length of the manubrium of the malleus, and the length of the head and neck, respectively. (E) *DG* is the total height of the incus, *EF* is the total width, and *FG* is the distance between long and short process. (F to H) Manual measurement of the total height of the incus, the total width of the incus, and the distance between long and short process, respectively. (I) *GI* is the total height of the stapes, and *HJ* is the length of stapes footplate. (J and K) Manual measurement of the total height of the stapes and the length of stapes footplate, respectively. (L) ∠*DGI* is incudostapedial joint angle. (M) Manual measurement of the incudostapedial joint angle.

Based on previous studies, literature, and clinical experience with ossicular-chain measurement [25–30], we defined the following measurement parameters, as shown in Fig. 2 and Table 1. For the malleus, 3 diameter parameters were measured: (a) total height (*AC*), defined as the maximum distance from the top of the head to the end of the manubrium; (b) length of the manubrium (*BC*), the maximum distance from the lateral process to the manubrium’s end; and (c) length of head and neck (*AB*), from the top of the head to the end of the lateral process. For the incus, we measured 3 diameter parameters: (a) total height (*DG*), the maximum distance from the incus body’s top to the long process tip; (b) total width (*EF*), the longest distance from the body to the short process tip; and (c) distance between long and short process (*FG*), the distance between their tips. For the stapes, 2 parameters were measured: (a) total height (*GI*) from the head to the footplate’s bottom, and (b) footplate length (*HJ*), the maximum footplate length. Additionally, the incudostapedial joint angle (∠*DGI*) and the volume of each ossicle were measured.

**Table 1.** The definition of measurement parameters of the ossicular chain

Measured parameters	Definition description
Total height of malleus	The maximum distance from the top of the head to the end of the manubrium of the malleus.
Length of manubrium of malleus	The maximum distance from the lateral process to the manubrium’s end of the malleus.
Length of head and neck	The maximum distance from the top of the head to the lateral process’s end of the malleus.
Total height of incus	The maximum distance from the incus body’s top to the long process tip.
Total width of incus	The maximum distance from the incus body to the short process tip.
Distance between long and short process	The maximum distance from the long process tip to the short process tip of the incus.
Total height of stapes	The maximum distance from the head to the footplate’s bottom.
Length of stapes footplate	The maximum footplate length.

The proposed algorithm identifies characteristic feature points on the reconstructed ossicular-chain surface and correlates them with defined anatomical landmarks to derive quantitative parameters. The processing pipeline comprises 3 sequential stages.

### Surface reconstruction and refinement

The initial segmentation results undergo subvoxel refinement using the Ricci curvature level set method to improve reconstruction fidelity. Subsequently, the Marching Cubes algorithm generates a triangular mesh representation from the volumetric data. To optimize surface quality, a windowed sinc function-based interpolation kernel performs low-pass filtering, effectively attenuating high-frequency noise while maintaining critical anatomical structures and geometric integrity. (For comprehensive technical details regarding the reconstruction methodology, see Ref. [31]).

### Feature point identification

The reconstructed surface undergoes differential geometric analysis, where Gaussian and mean curvature maps characterize local morphological features, while a minimum bounding box delineates spatial boundaries. Feature points are detected based on distinctive geometric signatures (e.g., prominent protrusions) and characteristic spatial distributions (e.g., terminal anatomical locations). This approach reliably identifies landmarks on the malleus and incus. For the structurally complex stapes—particularly in cases of footplate perforations or oblique orientation—principal components analysis (PCA) determines the dominant axis from its 2D projection. This axis constraint optimizes feature point localization accuracy across the stapes footplate.

### Anatomical parameterization

Using established spatial relationships among the ossicles, the detected feature points are mapped to their corresponding anatomical landmarks, enabling systematic computation of clinically relevant parameters.

### Manual measurement

Images in transverse, coronal, and sagittal views were displayed using RadiAnt DICOM Viewer software (Medixant, Poznan, Poland). Using the multiplane reconstruction function, we aligned the outermost intersection of the horizontal semicircular canal in the axial plane. The horizontal crosshair was then adjusted parallel to the canal in coronal and sagittal planes, obtaining standard views. Following the measurement parameters outlined in Fig. 2, we identified corresponding points on these images for manual measurements of distances and angles. During the manual measurement of the malleus, the intersection point of the cross-positioning lines was positioned at the malleoincudal joint within the cross-sectional plane. Next, the blue sagittal positioning line was adjusted to align with the direction of the short crus of the incus. Subsequently, the transverse positioning line was adjusted in the sagittal plane to ensure it is horizontally perpendicular to the head of the malleus. Finally, the maximum layer of the malleus will be displayed in the coronal plane. During the manual measurement of the incus, we began by positioning the intersection point of the cross-positioning lines on the incus within the cross-sectional plane. Next, the sagittal positioning line was aligned parallel to the direction of the short crus of the incus. Then, in the sagittal plane, the transverse positioning line was adjusted to coincide with the orientation of the short crus of the incus. This will allow the maximum section of the long crus of the incus to be displayed in the coronal plane, while the maximum section of the short crus of the incus will be visible in the sagittal plane. This setup facilitates the measurement of the height and width of the incus. Subsequently, in the coronal plane, the sagittal positioning line was adjusted to run parallel to the long crus of the incus. At this stage, both the long and short process of the incus will be simultaneously visible in the sagittal plane. During the manual measurement of the stapes, the long process of the incus was positioned at the center of the cross-positioning lines on the maximum display layer. Next, the transverse positioning line was adjusted to align it parallel to the direction of the stapes, thereby ensuring that the maximum layer of the stapes is displayed on the cross-sectional plane.

One observer manually measured the 3 ossicles in 47 of the 226 ears. Each parameter was measured 3 times, with the final measurement taken as the average of these results. Automated and manual measurements were then compared.

### Statistical analysis

Data analysis was conducted using SPSS (version 27.0; IBM Corp., Armonk, N.Y., USA). All measurements are reported as mean ± standard deviation (SD). The intraclass correlation coefficient (ICC) was used to evaluate the consistency of the results obtained from the 3 manual measurements. Comparisons between manual and automated measurements used a paired *t* test for normally distributed data and the Wilcoxon signed-rank test for non-normally distributed data. The ICC assessed consistency between manual and automated measurements, with values below 0.4 indicating poor consistency and values above 0.75 indicating high consistency. Automated measurement results were analyzed for differences across sex, side, and age groups. If data followed a normal distribution, an independent sample *t* test was used; otherwise, the Mann–Whitney rank *U* test was employed. *P* < 0.05 was considered statistically significant. In examining automated measurements across age groups, normality and homogeneity of variance were tested initially. If data met these criteria, a one-way analysis of variance (ANOVA) was performed; otherwise, a nonparametric rank-sum test was performed.

## Results

### Demographic results

A total of 140 patients (226 ears) were enrolled in this study, comprising 75 males and 65 females, with a mean age of 36.6 ± 14.2 years (Table 2). The dataset included 96 left and 130 right ears. A chi-square test was conducted on the number of people of different genders among the included ears, and the *P* value was 0.398. Since the *P* value is greater than 0.05, it indicates that the dataset is distributed according to gender. Then, the included ear data were grouped into 3 age groups [32]: there were 99 ears in the youth group (aged 18 to 30 years old), 101 ears in the middle-aged group (aged 31 to 60 years old), and 26 ears in the older adults group (aged 61 to 90 years old).

**Table 2.** Demographic information

Characteristic	Value
Number of ears	226
Mean age (mean ± SD)	36.6 ± 14.2
Sex (*n*) /%	
Female	75 (53.6)
Male	65 (46.4)
Laterality (*n*) /%	
Right	130 (57.5)
Left	96 (42.5)

### Segmentation results

**Fig. 3.**
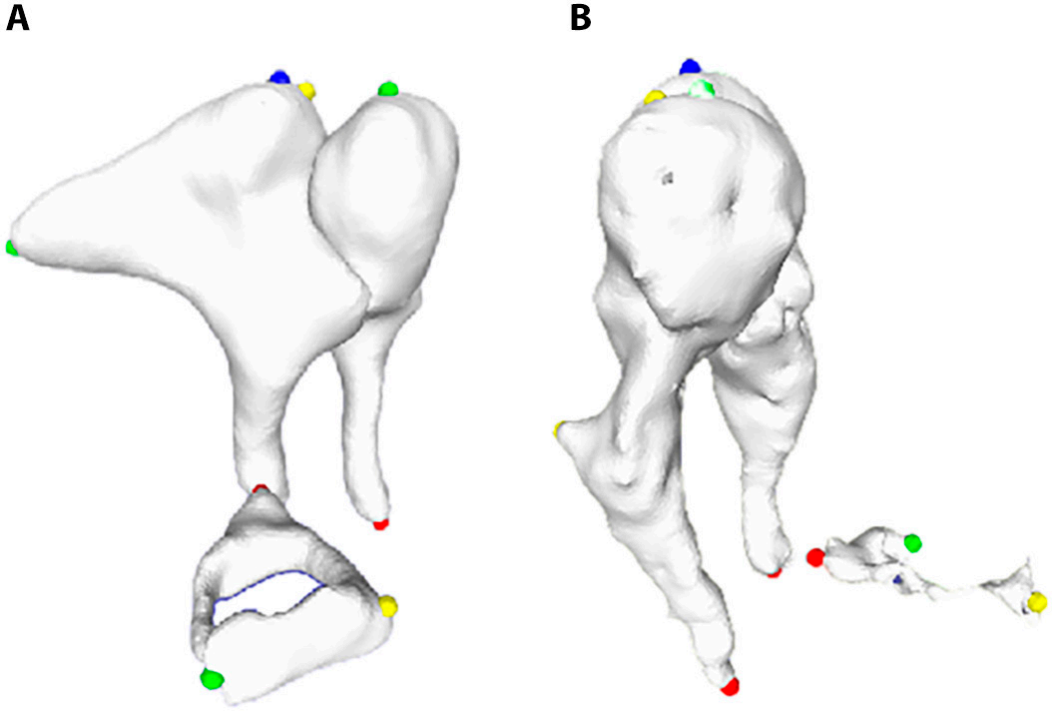
Results of 3D reconstruction of malleus, incus, and stapes. (A) The segmentation of malleus, incus, and stapes is complete. (B) The segmentation of malleus and incus is complete, while stapes segmented is incomplete.

From the original 2D CT images, automated segmentation masks, and subsequent 3D reconstructions, automated segmentation results were selected, excluding images with incorrect or incomplete segmentation of the 3 auditory ossicles. Complete separation of the malleus and incus was achieved in 226 ears, whereas the malleus, incus, and stapes were fully segmented in 47 ears. Fig. 3 illustrates examples of complete and incomplete 3D reconstructions of the auditory ossicles.

### Comparison of manual and automated measurement results

The same individual conducted repetitive measurements of 8 parameters, and the ICC values for the results obtained from the 3 manual measurements are presented in Table 3. All the ICC values are greater than 0.8, which indicates a high level of consistency among the results of the 3 manual measurements. In the subset of 47 ears with complete segmentation, automated and manual measurements of the malleus, incus, and stapes were compared (Table 4). The *P* values for all comparisons exceeded 0.05, indicating no statistically significant difference between manual and automated measurements. Additionally, all ICC values were above 0.75, demonstrating a high level of consistency between the 2 methods, thereby validating the automated measurements as comparable to manual measurements. The results of automatic measurement can replace those of manual measurement.

**Table 3.** The consistency of 3 manual measurement results

Measured parameters	ICC
Total height of malleus	0.970
Length of manubrium of malleus	0.968
Length of head and neck	0.987
Total height of incus	0.970
Total width of incus	0.807
Distance between long and short process	0.973
Total height of stapes	0.989
Length of stapes footplate	0.996

**Table 4.** Comparison of automated and manual measurement results (*n* = 47)

Measured parameters	Manual measurement	Automated measurement	*t* value	*P* value	ICC
Total height of malleus/mm	7.93 ± 0.39	7.91 ± 0.36	0.769	0.446	0.890
Length of manubrium of malleus/mm	4.37 ± 0.29	4.36 ± 0.28	0.452	0.653	0.988
Length of head and neck/mm	4.88 ± 0.31	4.89 ± 0.31	−1.659	0.104	0.990
Total width of incus /mm	4.99 ± 0.37	4.97 ± 0.33	0.615	0.541	0.831
Total height of incus /mm	6.57 ± 0.37	6.56 ± 0.38	0.398	0.693	0.978
Distance between long and short process/mm	5.57 ± 0.38	5.55 ± 0.39	1.721	0.092	0.985
Total height of stapes/mm	3.26 ± 0.20	3.25 ± 0.22	1.320	0.193	0.945
Length of stapes footplate/mm	2.63 ± 0.20	2.63 ± 0.15	−0.377	0.708	0.767

### Automated measurement of quantitative results

Automated measurements of the malleus and incus were conducted on all 226 ears, whereas only the 47 ears with complete stapes segmentation were included in the statistical analysis. Participants were initially grouped by sex, with measurement results for male and female groups presented in Table 5. Results indicated no significant differences in stapes footplate length, incudostapedial joint angle, or stapes volume between sexes (*P* > 0.05), whereas other measurements showed statistically significant sex-based differences. Further subgroup analysis was divided by sex and laterality (left versus right ear). The analysis showed statistically significant differences in the total height of the incus in females (*P* = 0.017) and the malleus volume in males (*P* = 0.037) between the right and left ears, with no significant differences observed in the other measurements. Finally, participants were categorized into 3 age groups: youth (18 to 30), middle-aged (31 to 60), and older adults (61 to 90), as shown in Table 6. No significant differences were found in any parameters across the age groups (*P* > 0.05), except for the volumes of the malleus and incus (*P* = 0.015, *P* = 0.031) . This suggests that the morphology of adult auditory ossicles remains stable and fully developed across these age ranges.

**Table 5.** Comparison of automated measurement results between sex and laterality (malleus, incus: *n* = 226; stapes and angle: *n* = 47)

Measured parameters	Female	Male	*t/Z* value	*P* value	Female				Male			
					Right	Left	*t/Z* value	*P* value	Right	Left	*t/Z* value	*P* value
Total height of malleus/mm	7.80 ± 0.48	8.07 ± 0.44	−5.103	<0.001	7.73 ± 0.56	7.89 ± 0.34	−1.184	0.236	8.07 ± 0.33	8.08 ± 0.58	−0.546	0.585
Length of manubrium of malleus/mm	4.28 ± 0.39	4.55 ± 0.53	−4.930	<0.001	4.24 ± 0.43	4.33 ± 0.33	−0.849	0.396	4.58 ± 0.62	4.50 ± 0.35	−0.098	0.922
Length of head and neck/mm	4.86 ± 0.50	4.97 ± 0.36	−3.137	0.002	4.83 ± 0.61	4.89 ± 0.30	−0.524	0.600	4.95 ± 0.31	5.00 ± 0.43	−1.336	0.181
Total height of incus/mm	6.45 ± 0.58	6.71 ± 0.36	−4.303	<0.001	6.34 ± 0.72	6.59 ± 0.31	−2.385	0.017	6.72 ± 0.35	6.68 ± 0.38	−0.353	0.724
Total width of incus/mm	4.91 ± 0.37	5.17 ± 0.58	−4.237	<0.001	4.91 ± 0.42	4.91 ± 0.29	−0.571	0.568	5.13 ± 0.50	5.23 ± 0.68	−0.209	0.835
Distance between long and short process/mm	5.58 ± 0.32	5.63 ± 0.62	−2.342	0.019	5.54 ± 0.35	5.62 ± 0.28	−1.234	0.217	5.69 ± 0.39	5.53 ± 0.85	−0.353	0.724
Total height of stapes/mm	3.18 ± 0.21	3.38 ± 0.20	−2.80	0.005	3.18 ± 0.23	3.19 ± 0.15	0.087	0.931	3.40 ± 0.20	3.19 ± 0.04	−1.460	0.166
Length of stapes footplate/mm	2.61 ± 0.14	2.67 ± 0.17	−1.286	0.205	2.63 ± 0.14	2.57 ± 0.14	−1.119	0.272	2.70 ± 0.16	2.46 ± 0.20	−2.017	0.063
Incudostapedial joint angle/(°)	96.83 ± 6.03	95.79 ± 5.17	0.587	0.560	96.90 ± 6.58	96.66 ± 4.75	−0.099	0.922	96.06 ± 5.20	93.95 ± 6.55	−0.527	0.606
Volume of malleus/mm^3^	12.43 ± 1.52	13.48 ± 1.61	−5.004	<0.001	12.25 ± 1.57	12.65 ± 1.45	−1.569	0.117	13.85 ± 1.56	13.23 ± 1.61	2.111	0.037
Volume of incus/mm^3^	13.33 ± 1.82	14.29 ± 1.73	−4.056	<0.001	13.20 ± 1.92	13.50 ± 1.70	−0.817	0.416	14.12 ± 1.73	14.55 ± 1.70	1.386	0.168
Volume of stapes/mm^3^	1.64 ± 0.30	1.78 ± 0.40	−1.395	0.170	1.66 ± 0.21	1.60 ± 0.46	0.351	0.733	1.81 ± 0.42	1.59 ± 0.01	1.946	0.074

**Table 6.** Comparison of automated measurement results at different ages (malleus, incus: *n* = 226; stapes and angle: *n* = 47)

Measuring parameters	Youth group	Middle-aged group	Older adult group	*P* value
Total height of malleus/mm	7.87 ± 0.49	8.01 ± 0.49	8.04 ± 0.29	0.102
Length of manubrium of malleus/mm	4.35 ± 0.40	4.48 ± 0.59	4.49 ± 0.26	0.114
Length of head and neck/mm	4.85 ± 0.43	4.97 ± 0.46	4.99 ± 0.24	0.054
Total height of incus/mm	6.61 ± 0.36	6.53 ± 0.62	6.77 ± 0.32	0.068
Total width of incus/mm	4.96 ± 0.40	5.11 ± 0.59	5.15 ± 0.54	0.075
Distance between long and short process/mm	5.63 ± 0.35	5.55 ± 0.65	5.73 ± 0.35	0.211
Total height of stapes/mm	3.20 ± 0.22	3.34 ± 0.20	3.19 ± 0.24	0.178
Length of stapes footplate /mm	2.66 ± 0.17	2.61 ± 0.14	2.60 ± 0.70	0.457
Incudostapedial joint angle/(°)	97.46 ± 6.28	96.24 ± 5.15	92.64 ± 3.45	0.227
Volume of malleus/mm^3^	12.67 ± 1.59	13.24 ± 1.78	13.35 ± 1.14	0.015
Volume of incus/mm^3^	13.62 ± 1.88	13.94 ± 1.77	14.46 ± 1.75	0.031
Volume of stapes/mm^3^	1.74 ± 0.40	1.62 ± 0.26	1.69 ± 0.26	0.677

## Discussion

This study developed a systematic method for automated segmentation and measurement of the ossicular chain, analyzing results across sex, side, and age groups. Findings indicate that automated measurements closely match manual measurements and can effectively replace them, with sex-based anatomical differences also observed in ossicular-chain parameters. Consequently, surgeons should consider these anatomical differences during ossicular-chain reconstruction surgery to ensure tailored surgical approaches.

In a related study, Javia and Saravanan [13] measured the malleus using a Vernier caliper and reported average measurements in males for total height, manubrium length, and head–neck length as 7.88 ± 0.45 mm, 4.59 ± 0.45 mm, and 5.06 ± 0.38 mm, respectively. In females, corresponding measurements were slightly smaller: 7.63 ± 0.48 mm, 4.48 ± 0.37 mm, and 4.95 ± 0.25 mm. These measurements closely align with the values obtained in our study, with a minimal difference ranging from 0.04 to 0.20 mm. This consistency highlights the statistically significant difference in malleus dimensions between sexes, a finding that reinforces our results. Singh et al. [27], who measured 60 mallei using a digital Vernier caliper, found no significant difference between left and right ears in any of the 3 measured parameters, which is consistent with the study’s results. The mean (SD) length of the manubrium in the 35 cases was 4.39 (+0.34) mm, comparable to the 4.42 (+0.48) mm calculated by the proposed algorithm.

For the incus, Unur et al. [33] measured the mean total height and width as 6.47 and 4.88 mm, respectively, aligning well with the results of this study. Additional studies [34] also indicated no statistically significant differences in incus measurements between sides, although we observed a significant side-based difference in the total height of the incus in females, which may be attributed to our relatively small sample size.

Rathava et al. [35] measured the stapes with the mean height of 3.33 ± 0.25 mm (ranging from 2.86 to 3.9 mm) and an average footplate length of 2.78 ± 0.15 mm (ranging from 2.41 to 3.11 mm). Similarly, Tang et al. [12] measured stapes height in temporal bone U-HRCT scans, reporting a mean of 3.48 ± 0.33 mm. Our study’s automated measurements of the stapes height (3.25 ± 0.22 mm) and footplate length (2.63 ± 0.15 mm) are consistent with these values, affirming the reliability of our automated approach.

Our analysis also included age-based statistical evaluations for adults aged 18 years and older. Findings show that ossicular-chain parameters remained stable across age groups, with the exception of the volume of the malleus and incus. Previous studies [36] suggest that middle ear ossicles reach the adult size early in life, maintaining stable morphology and parameters into adulthood. Therefore, in clinical practice, heightened care is advised when assessing hearing in newborns to ensure accurate, reliable results. The findings of the present study are similar to the conclusions derived from previous studies.

The measurement of the morphological parameters of the ossicular chain is of significant clinical importance for disease diagnosis, otological surgical planning, and the preparation of personalized ossicular-chain prostheses. In clinical diagnosis, physicians primarily determine whether the ossicular chain is normal or abnormal based on its morphology and specific measured parameters. Li et al. [37] found statistically significant differences in the length of the manubrium of the malleus and the angle of the incudostapedial joint among congenital aural atresia, congenital aural stenosis, and normal ears. The differences in measured parameters help physicians diagnose a variety of diseases more effectively. For surgeries such as ossicular-chain reconstruction, accurate preoperative assessment is crucial. The morphological features of normal and malformed ossicles described in the report may assist surgeons in evaluating the ossicles in their patients before performing ossiculoplasty. Precise preoperative measurements enable surgeons to better understand the anatomical structure of patients before the operation, thereby facilitating the formulation of a more accurate surgical plan. In the preparation of ossicular-chain prostheses, variations in the angle of the incudostapedial joint significantly influence the selection of prosthesis length [38]. Incorrect prosthesis sizing and crimping are frequently associated with stapedectomy failure. If the deviation of the incudostapedial joint angle from 90° is as little as 4.5°, a change in prosthesis size is necessary [38]. Based on the measured parameters of the ossicular chain, personalized prosthesis preparation can be effectively achieved.

Furthermore, the present study employed U-HRCT, which results in clearer images and is based on the automatic measurements of 3D results that are more accurate and consistent. High-resolution U-HRCT imaging offers clear visualization of the ossicular chain’s morphology, providing valuable data for individualized surgical planning. This approach not only supports surgical precision and better outcomes but also minimizes the potential hearing impact and enables the detection of lesions based on high-resolution data. However, a limitation of the study is that measurement results are affected by segmentation results. Especially when dealing with complex morphological structures, such as the stapes, the low precision of automatic stapes segmentation results in incomplete automatic segmentation of certain stapes. For example, the stapes footplate might be missing. These challenges impact the selection of key feature points on the stapes, ultimately hindering the automatic measurement of parameters. In future work, we will concentrate on improving the segmentation accuracy of the stapes. This enhancement will provide a solid foundation for subsequent automatic measurements and medical diagnoses. In addition, the manual annotations in this study were carried out in a single-blind manner both during the annotation and review processes. The quality of annotation results significantly depends on the experience of the reviewers. This, in turn, directly affects the performance of automatic segmentation algorithms. Therefore, we plan to optimize the annotation methods in the next phase. Another limitation is that the study employed data from one institution, and only the U-HRCT images with 0.1-mm voxel resolution were considered. Therefore, variations in scanner settings and resolutions will decline the accuracy of segmentation, and septically lower resolution images will affect the topologic completeness of the stapes. We cannot automatically measure some quantitative parameters based on the incomplete segmentation results. The topologic completed stapes segmentation should be further investigated in our future work. Due to the limitation of hospitals that have installed U-HRCT equipment, there is currently no data foundation for multi-center verification. We will promote this verification work in the future.

## Conclusion

This study utilized automated segmentation and measurement techniques on U-HRCT images to analyze the ossicular chain. Results showed statistically significant differences between sexes for all measured parameters, except for the stapes footplate length, incudostapedial joint angle, and stapes volume. These findings offer valuable insights into biological sex differences, supporting the detection of abnormalities and aiding in disease diagnosis based on the anatomy of the normal ossicular chain.

## Data Availability

The data used to support the findings of this study are available from the corresponding author upon request.
